# Ultrasonication affects the melatonin and auxin levels and the antioxidant system in potato *in vitro*

**DOI:** 10.3389/fpls.2022.979141

**Published:** 2022-09-29

**Authors:** Georgina Pesti-Asbóth, Piroska Molnár-Bíróné, Ildikó Forgács, Judit Remenyik, Judit Dobránszki

**Affiliations:** ^1^ Institute of Food Technology, Faculty of the Agricultural and Food Science and Environmental Management, University of Debrecen, Debrecen, Hungary; ^2^ Centre for Agricultural Genomics and Biotechnology, Faculty of the Agricultural and Food Science and Environmental Management, University of Debrecen, Nyíregyháza, Hungary

**Keywords:** redox homeostasis, endogenous melatonin, glutathione, lipid peroxidation, indole-3-acetic acid

## Abstract

Melatonin is an ancient hormone whose physiological effects have been extensively studied in animals and human. We now know that it also plays a prominent role in the growth and development of plants. In our present experiment, the relationship between endogenous melatonin and the antioxidant system was investigated in potato plant grown *in vitro*. Changes in redox homeostasis under ultrasound stress were examined. The concentration of small molecule antioxidants and enzymes of the three-level antioxidant pathway was measured. ELISA method was used to determine the melatonin levels in plant tissues at each growth stage (0 h, 24 h, 48 h, 1 week, and 4 weeks after subculturing the explants) both in control and ultrasound-treated plants. Ultrasound stress activated the three-level defense system and decreased the endogenous melatonin levels. Melatonin was able to provide protection against membrane damage caused by drastic ultrasound treatment. Melatonin at the heart of the redox network is a key component regulating various biochemical, cellular, and physiological responses. It has a dual role, as it is able to act both as a growth regulator and an antioxidant. A close relationship was evidenced between the plant hormone indole-3-acetic acid and melatonin and ascorbic acid.

## 1 Introduction

Melatonin (MT) is a long-known (since the 1950s) hormone, and several research studies that have been published point out that it takes part, either directly or indirectly, in the regulation of almost every physiological process, including the circadian rhythm (Dijk and Czeisler, 1994). The new miracle of this old hormone was the discovery of its synthesis in plants, and like its multiple roles in vertebrates, it is also essential in them (Cassone, 1998; Arnao and Hernández-Ruiz, 2013; Arnao and Hernández-Ruiz, 2014; Arnao and Hernández-Ruiz, 2015; Battacharya and Jha, 2020).

Several research groups have started to study the effects of exogenous MT on the agricultural crops. It was studied in terms of how it can raise biomass by increasing stress tolerance (Erland et al., 2015). Experiments have proven that it inhibits chlorophyll degradation in leaves or their ageing (Erland et al., 2015). MT can enhance rooting, root regeneration, and the adaptation to different abiotic stress factors, thus tolerating salt stress and water shortage (Li et al., 2017). These effects directly contributed to improved yields and increased food production. Most of the world face malnutrition and increased food demand with low resources, so it is important to know how MT can play a significant role in improving crop yield. However, it is very important to investigate the possible and potential role of MT in detail, including with model systems such as *in vitro* plant tissue culture. This is important because MT has several functions (Arnao and Hernández-Ruiz, 2019), so it cannot be questioned that it may have a toxic concentration both on plants and on the other members of the food chain. We do not have yet convincing knowledge of its effect on bacteria and fungi, the microflora of soil and animals.

Although more and more data are available on the growth-stimulating effect of exogenous MT, little is known about its regulatory role in the presence and accumulation of endogenous MT. This is understandable since it is present in different amounts in different plant species and even varieties, but it is also known that the amount is different in different parts of the plant within a plant and depends also on the developmental stage (Van Tassel et al., 2001; Arnao and Hernández-Ruiz, 2015). Moreover, it is known that its concentration varies depending on the time of day (Arnao and Hernández-Ruiz, 2015). There is likewise the lack of a uniform, accepted test protocol facilitating the determination of endogenous melatonin levels.

In our present study, stress conditions were induced by different ultrasound treatments. Ultrasound is an abiotic stressor that can also be used to modify plant growth and development *in vitro*, and its use can be incorporated into existing micropropagation techniques easily (Teixeira da Silva and Dobránszki, 2014; Teixeira da Silva et al., 2020). The aim of our present experimental work was to study the mechanisms which help to understand the role of MT under stress conditions and its relationship with indole-3-acetic acid (IAA) metabolism. How can MT coordinate the growth and improve the stress response of plants *in vitro*? What cascade processes were induced by ultrasound stress, and how does the endogenous MT-IAA concentration change? How can MT protect photosynthesis *via* supporting chlorophyll synthesis during adaptation to abiotic (ultrasound) stress?

## 2 Materials and methods

### 2.1 Plant material and ultrasonication of *in vitro* plant explants

The plant material (*Solanum tuberosum* L. cv. Desirée, 4-week-old *in vitro* plantlets) and growing conditions *in vitro* were identical as described earlier in Teixeira da Silva et al. (2020). The *in vitro* single-node segments of stems excised from 4-week-old plantlets were ultrasonicated at 35 kHz, 20°C, and 70 W for 0 (control), 20, and 30 min by Elmasonic X-tra 30 H ultrasonicator and then cultured on Murashige and Skoog (1962) medium (MS medium) as described in detail earlier (Teixeira da Silva et al., 2020). In short, ultrasonication occurred in the ultrasonicator in 50-ml glass beakers, each containing 90 explants immersed in 40 ml liquid MS medium. The beakers were surrounded by distilled water in the ultrasonicator, and the temperature of the water was kept constant at 25°C. The explants were placed in 400-ml glass jars containing 50 ml of MS medium and 30 explants; in total, 750 explants were used for each treatment. The plant cultures were grown *in vitro* for 4 weeks at 22 ± 2°C under a 16-h photoperiod at a photosynthetic photon flux density of 63.5 μmol m^−2^ s^−1^.

### 2.2. Sampling times and measurements of morphological parameters and chlorophyll content

Plant samples were taken at 0 h, 24 h, 48 h, 1 week, and 4 weeks after ultrasonication, and they were stored at -80°C until biochemical analysis. For sampling, at least 90 explants (at 0, 24, and 48 h) or plantlets (at 1 and 4 weeks) were used from three different jars.

At the end of the *in vitro* subculture (at 4 weeks), the length and fresh weight of shoots and roots and the number of nodes per plantlet were measured. The chlorophyll a (*chl a*), chlorophyll b (*chl b*), and *chl a* + *chl b* contents in shoots of 4-week-old plantlets were determined spectrophotometrically, and the ratio of *chl a*/*chl b* was calculated as described earlier (Dobránszki and Mendler-Drienyovszki, 2014; Dobránszki et al., 2017). For morphological measurements and chlorophyll content analysis, plant samples taken from five jars, each of which contained 30 explants/plantlets, were used.

### 2.3 Biochemical analyses

#### 2.3.1 Sample preparation

Three replicates of each plant sample were used for sample preparation, each containing 250 mg of plant material. Frozen (plant) samples were homogenized with mortar and pestle under liquid nitrogen and extracted in 3 ml of ice-cold 50 mmol sodium phosphate buffer. The received solution was centrifuged at 10,000 × *g* at 4°C for 20 min. The supernatant was collected and stored at -20°C until the experiments. Through measuring the glutathione (GSH), glutathione reductase (GR), superoxide dismutase (SOD), ascorbic acid peroxidase (APX), IAA, glutathione synthetase (GSS), and ascorbic acid (AA) levels, absorbance was determined by a BMG Labtech SpectroStar Nano microplate reader.

Moreover, 200 mg of samples was frozen at -70°C and lyophilized to determine the lipid-soluble antioxidant capacity (ACL) and the water-soluble antioxidant capacity (ACW). After dehydration, the samples were ground to fine powder. To measure ACL and ACW, 25–25 mg of dry powder was dissolved in 1 ml methanol and 1 ml dH_2_O, respectively, during electromagnetic stirring for 60 s at 25°C. The homogenates were centrifuged at 10,000 × *g* at room temperature for 5 min. The supernatant was collected and stored at -20°C until the measurement. ACL and ACW were detected with Analytikjena PhotoChem.

#### 2.3.2 Measurements

Commercially available kits were used for the determination of catalase (CAT; Abnova, Taipei, Taiwan), SOD (ACW:849-60002-0; SOD: SIGMA: S7571; Analytikjena AG, Jena, Germany), GSH (kit number: 703002 Cayman Chemical Ann Arbor, MI, USA), GR (kit number: ab83461; Abcam plc., Cambridge, UK), GST (Abcam plc.), APX (MyBiosource, Inc., San Diego, CA, USA), ACW (kit number: 846-60002-0; Analytikjena AG), ACL (kit number: 849-60004-0; Analytikjena AG), AA (kit number: ab65346; Abcam), indol-3-aceic acid (kit number: CEA737Ge; Cloud-Clone Corp. Huston, Texas), melatonin (kit number: ab213978; Abcam plc., Cambridge, UK), glutathione synthase (kit number: MBS9373764; MyBioSource, Inc., San Diego, CA, USA). The reaction mixtures in each measurement were prepared, and then calculations were performed as described in each protocol book.

##### 2.3.2.1 Determination of SOD

SOD was determined with a commercially available ACW kit (ACW: 849-60002-0; SOD: SIGMA: S7571; Analytikjena AG, Jena, Germany). The SOD of samples ensures the removal of superoxide anion radicals ( ${\text{O}}_{2}^{•-}$ ) formed by the auto-oxidation of hemoglobin. Furthermore, 1,500 µl R1, 1,000 µl R2, and 25 µl R3 reagents of ACW kit were mixed for making blanks. Using at least five points with the standard of the SOD enzyme, a calibration was made between 0.02112 and 0.2112 nmol. For measurements, we used the supernatant of the samples. Moreover, 1,490 µl of R1, 1,000 µl of R2, 25 µl of R3, and 10 µl of the samples were included in the working solution. The results generated by PhotoChem were compared with the standard equation and re-counted to units per milliliter. The SOD activity of the samples was expressed in units per milliliter.

##### 2.3.2.2 Determination of reduced glutathione and oxidized glutathione

The concentration of reduced glutathione and oxidized glutathione was determined using a commercially available assay kit (kit number: 703002 Cayman Chemical Ann Arbor, Michigan). In the assay, 5,5′-dithio-bis(2-nitrobenzoic acid) (DTNB) and GSH react and generate a yellow product (2-nitro-5-thiobenzoic acid). Under the assay conditions, GSSG was reduced, producing 2 mEq of GSH. The kit reagents were dissolved according to the “reagent preparation” chapter. The standard curve was prepared as described in the kit. Then, 50 µl of standards was added to the designated wells on the plate. Moreover, 50 µl of each sample was added to the sample wells. The assay cocktail contained 117.2 µl MES buffer, 4.70 µl cofactor mixture, 22 µl enzyme mixture, 24 µl dH_2_O, and 4.67 µl reconstituted DTNB per well. In addition, 150 µl assay cocktail was added to each of the wells containing standards and samples. The plate was incubated at 25°C for 25 min in the dark. The measurement was started after the incubation immediately. The concentration of GSH and GSSG was determined by measuring the absorbance colorimetrically at 405 nm. The sample GSH concentration was compared with a GSH standard curve, and the concentration of GSH was expressed as plant GSH concentration (in µM). The sample GSSG concentration was compared with the GSSG standard curve, and the concentration of GSSG was expressed as plant GSSG concentration (in µM).

##### 2.3.2.3 Determination of glutathione reductase

The GR activity was determined using a commercially available assay kit (kit number: ab83461; Abcam plc., Cambridge, UK). In this assay, GR forms GSH from GSSG; then, while GSH reacts with DTNB, 2-nitro-5-thiobenzoate anion (TNB^2-^) is generated. By measuring the change in absorbance at 405 nm, the GR activity can be determined. The kit reagents were dissolved as described in the protocol book. The samples were pre-treated first. Next, 5 µl 3% H_2_O_2_ was added to 100 µl of each sample. The samples were incubated at 25°C for 5 min. Then, 5 µl of CAT was added to the samples, and the samples were incubated at 25°C for another 5 min. After the pre-treating procedure, 50 µl of the pre-treated samples was added into the sample wells. The standard curve was prepared as described in the kit, and 50 µl of the diluted standard solution was added into the correct wells. The reaction mixture was prepared as described in the protocol book. The mix contained 40 µl GR assay buffer, 2 µl DTNB solution, 2 µl NADPH solution, and 6 µl GSSG solution per well, except the standard wells. Furthermore, 50 µl of the reaction mixture was added to the samples. The microplate reader was set to take one reading every 5 min for 30 min for GR activity measurement. The measurement was started immediately after the addition of the reaction mixture. The kinetics of the reaction was read to ensure that *A*_1_ and *A*_2_ were in a linear reaction range, and Δ*A*_405nm_ = *A*_2_ - *A*_1_ (*A*_2_ is the absorbance data in the second reading, and *A*_1_ is the absorbance data in the first reading). The TNB standard curve was plotted, and Δ*A*_405nm_ was applied to this curve to obtain ΔB nmol of TNB. The obtained result was substituted into the equation described in the kit’s instruction:

$\text{GR activity}=\frac{\Delta \text{B}}{\left({\text{T}}_{2}-{\text{T}}_{1}\right)\;0.9\text{V}}\text{ D}=\text{nmol}/\text{min}/\text{mL}=\text{mU}/\text{mL}$ [ΔB is the TNB amount from the TNB standard curve (in nmol)]. *T*_1_ is the time of the first reading (*A*_1_) (in min). *T*_2_ is the time of the second reading (*A*_2_) (in min). *V* is the pre-treated sample volume added into the reaction well (in ml); 0.9 is the sample volume change factor during the sample pre-treatment procedure; *D* is the dilution factor, as suggested in the kit’s description. The GR activity was expressed as plant GR activity (in nmol/min/ml = mU/ml).

##### 2.3.2.4 Determination of GPX

The glutathione peroxidase (GPX) activity was determined using a commercially available assay kit (kit number: ab102530; Abcam plc.). In this assay, GPX generates GSSG from GSH during H_2_O_2_ reduction, and that generated GSSG reduced back to GSH by GR during the consumption of nicotinamide adenine dinucleotide phosphate (NADPH). The NADPH reduction is proportional to the GPX activity and can be measured colorimetrically at 340 nm. The kit reagents were dissolved as described in the protocol book. The standard curve was prepared as described in the kit, and 90 µl of the diluted standard solutions was added into the correct wells. Then, 50 µl of samples, positive control, and reagent control was added to the plate into the correct well. The reaction mixture was as described in the protocol book. The mix contained 33 µl assay buffer, 3 µl 40 mM NADPH solution, 2 µl GR solution, and 2 µl GSH solution per well except the standard wells. Then, 40 µl of the reaction mix was added to the samples, positive control, and reagent control. The plate was incubated at room temperature for 15 min. After incubation, the absorbance was measured. If the absorbance value of the samples was lower than 1.0, then 1 µl of 40 mM NADPH was added to the sample. Moreover, 1 µl of 40 mM NADPH gives a ~0.5 increase of absorbance at 340 nm. After this step, 10 µl of cumene hydroperoxyde was added to the samples, positive control, and reagent control wells to start the GPX reaction. The microplate reader was set to take one reading every 5 min for 30 min for GR activity measurement. The measurement was started immediately after the addition of the cumene hydroperoxide at Δ*A*_340nm_ [Δ*A*_340nm_ = (sample *A*_1_ – sample *A*_2_) - (reagent control *A*_1_ - reagent control *A*_2_) (*A*_1_ is the absorbance value in the first reading, and *A*_2_ is the absorbance data in the second reading)]. The NADPH standard curve was plotted, and Δ*A*_340nm_ was applied to this curve to obtain ΔB nmol of NADPH. The obtained result was substituted into the equation:

$\text{GPX activity}=\frac{\Delta \text{B}}{\left({\text{T}}_{2}-{\text{T}}_{1}\right)\text{ V}}\text{ D}=\text{nmol}/\text{min}/\text{mL}=\text{mU}/\text{mL}$ [ΔB = NADPH amount that was decreased between *T*_1_ and *T*_2_ (in nmol), *T*_1_ = time of the first reading (*A*_1_) (min), *T*_2_ = time of second reading (*A*_2_) (min), *V* = pre-treated sample volume added into the reaction well (ml), and *D* = sample dilution factor]. The NADPH standard curve was plotted, and the equations suggested by the kit were used. The activity of GPX was expressed as a plant GPX activity (mU/ml).

##### 2.3.2.5 Determination of glutathione-S-transferase

The glutathione-S-transferase (GST) activity was determined by a commercially available kit (Abcam plc.). The assay is based on the reaction between GSH and 1-chloro-2,4-dinitrobenzene (CDNB), of which the reaction depends on the presence of the active GST under certain conditions. The kit reagents were dissolved as described in the protocol book. Then, 50 µl of the samples, diluted positive control, and negative control was added into the correct wells. The reaction mixture was as described in the protocol book. The mix contained 49 µl GST assay buffer and 1 µl GST substrate (CDNB) solution per well. In addition, 50 µl of the reaction mixture was added to each well. The microplate reader was set to take one reading every 2 min for 16 min for GST activity measurement. The measurement was started immediately after the addition of the GST reaction mix. The changes of the absorbance were measured at 340 nm.


$$\Delta A340nm=\frac{\Delta A340\text{ (time2)}-\Delta A340\text{ (time1)}}{\text{Time }2\text{ (min)}-\text{Time }1\text{ (min)}}\;$$

(*A*_1_ is the absorbance value in the first reading, and *A*_2_ is the absorbance data in the second reading) was determined for all samples. The obtained result was substituted into the equation:

$\text{GST activity}=\frac{\Delta A340 nm}{\text{(0}.0096)\text{ (0}.2893)\text{ (D/A)}}\;$ [0.0096 µmol^-1^ cm^-1^ is the extinction coefficient of the glutathione-DNB adduct, *A* = sample volume added into the reaction well (ml), *D* = sample dilution factor, *V* = sample volume added to the well (ml), and 0.2893 cm is the light path of the 0.1 ml reaction volume in a Greiner Bio One 655101 96-well plate (cm)]. The GST activity of the samples was expressed as plant GST activity (in mU/ml).

##### 2.3.2.6 Determination of APX

APX was determined using a commercially available kit (MyBiosource, Inc., San Diego, CA, USA). The kit is based on double-sandwich ELISA where the substrate (TMB) finally turns into a yellow product and the depth of the yellow color was measurable at 450 nm and positively correlated with APX activity. The kit reagents were dissolved as described in the protocol book. The standard curve was prepared as described in the kit, and the diluted standard solutions were added into the correct wells. First, samples, blank, and different concentrations of standards were added to the corresponding wells (100 µl for each well). The reaction wells were sealed with adhesive tapes and incubated at 37°C for 90 min. After this step, the plate was washed three times with 350 µl diluted washing buffer. Thereafter, 100 µl of biotinylated plant APX antibody liquid was added to each reaction well. The reaction wells were sealed with adhesive tapes, and the plate was incubated at 37°C for 60 min. After incubation, the plates were washed as described previously. Then, 100 µl of enzyme–conjugate liquid was added to each well, except the blank wells. The reaction wells were sealed with adhesive tapes and incubated at 37°C for 30 min. The plate was washed five times as described. Then, 100 µl of Colour Reagent was added to each well. The reaction wells were sealed with adhesive tapes and incubated at 37°C for 30 min. In the last step, 100 µl of Colour Reagent C was added to each well, and the absorbance was measured at 450 nm. The concentration of APX in the samples was determined by comparing the absorbance values of the samples to a standard curve. The APX concentration was expressed as plant APX concentration (in ng/ml).

##### 2.3.2.7 Determination of the antioxidant capacity of water-soluble compounds

ACW was determined using a commercially available ACW kit (kit number: 846-60002-0; Analytikjena AG). Radicals are generated photo-chemically by UV irradiation of a photosensitizer compound. The PhotoChem measures the inhibition of radicals by the sample ACW content.

The ACW kit contained R1, R2, R3, and R4 reagents. R1 and R2 were ready for use and stored at 2–8°C. R3 was lyophilized and stored at -20°C until use. Furthermore, 750 µl of R2 reagent was added into R3 and was ready to use. A stock solution was made from R4 reagent; 490 µl of R1 reagent and 10 µl of H_2_SO_4_ were added into R4 reagent. Ten-fold dilutions were done from the stock solution (10 µl of R4 stock solution + 990 µl of R1 solution). First, the blank was made: 1,500 µl of R1, 1,000 µl of R2, and 25 µl of R3 were mixed. The calibration was made between 0, 1, and 2 nmol (at least five points) with L-ascorbic acid standard. Moreover, 25 mg of the lyophilized sample was dissolved in dH_2_O and mixed for 90 s after the sample was centrifuged at 10,000 rpm for 5 min. The supernatant of the prepared samples was used for the measurement. The working solution contained 1,490 µl of R1, 1,000 µl of R2, 25 µl of R3, and 10 µl of the samples. The calculation for the lyophilized sample was as follows:


$$\text{Concentration}=\frac{\text{(results) (dilution) (176}.13)}{\text{(sample volume) (used lyophilized quantity)}}\;$$

The result by the PhotoChem; 176.13: vitamin C molecular weight; sample volume: pipetted volume (in µl), lyophilized sample quantity (in mg). The final result was added in microgram per milligram (µg/mg) ascorbic acid equivalent.

##### 2.3.2.8 Determination of antioxidative capacity of lipid compounds

The antioxidative capacity of lipid compounds (ACL) was determined using a commercially available ACL kit (kit number: 849-60004-0; Analytikjena AG). The methodology is similar as that which was used for the ACW test. The ACL kit contained R1, R2, R3, and R4 reagents. R1 and R2 were ready for use and stored at 2–8°C. R3 was lyophilized and stored at -20°C until use. Moreover, 750 µl of R2 reagent was added into R3 and was ready to use. The stock solution was made from R4 reagent, 500 µl of R1 reagent was added into R4 reagent, and there were 10-fold dilutions obtained from the stock solution (10 µl of R4 stock solution + 990 µl of R1 solution. First, the blank was made: 2,300 µl of R1, 200 µl R2, and 25 µl R3 were mixed. The calibration was made between 0.2 and 3 nmol (at least five points) with trolox standard. Then, 25 mg of the lyophilized sample was dissolved in 1 ml of methanol and mixed for 90 seconds after the sample was centrifuged at 10,000 × *g* for 5 min. The supernatant of the prepared samples was used for the measurement. The working solution contained 2,290 µl of R1, 200 µl of R2, 25 µl of R3, and 10 µl of the samples. The calculation for lyophilized sample is as follows:


$$\text{Concentration}=\frac{\text{(results) (dilution) (250}.3)}{\text{(sample volume) (used lyophilized quantity)}}\;$$

The result by PhotoChem; 250.3: trolox molecular weight; sample volume: pipetted volume (in µl), lyophilized sample quantity (in mg). The final result was added in µg/mg trolox equivalent.

##### 2.3.2.9 Determination of ascorbic acid (vitamin C)

The ascorbic acid (AA) concentration was determined using a commercially available assay kit (kit number: ab65346; Abcam plc.). In this assay, a proprietary catalyst oxidizes AA to produce products that interact with the AA probe, generating color. The kit reagents were dissolved as described in the protocol book. The standard curve was prepared as described in the kit, and the diluted standard solutions were added into the correct wells (120 µl); 120 µl of the blank and samples, respectively, was added to the correct well. The reaction mix was prepared according to the protocol book. Moreover, 50 µl of the reaction mix was added to each well. The color was developed within 3 min and stable for an hour. Absorbance was measured at 570 nm. An AA standard curve was plotted, and the equation $\text{Concentration}=\frac{\text{As}}{\text{Sv}}\;$ (where As is ascorbic acid amount from the standard curve, and Sv is the sample volume added in the sample wells) was used as suggested in the kit’s description. The results were multiplied by the molecular weight of AA (176.12 g). The AA concentration of samples was expressed as plant AA concentration (in ng/µl) [1].

##### 2.3.2.10 Determination of melatonin

Melatonin was determined using a commercially available assay kit (kit number: ab213978; Abcam plc.). The determination of melatonin is independent of species. All reagents and samples were prepared as described in the protocol book. To determine the melatonin concentration, 500 mg of sample was ground in liquid nitrogen. The dried and ground samples were dissolved in 125 µl of stabilizer solution, and then 750 µl cold ethyl acetate was added. The sample solutions were vortexed for 30 s and were placed on ice for 3 min. After this, they were vortexed again as mentioned previously and were placed on ice for 2 min. The solutions were placed in ultrasonic bath for 5 min. All samples were centrifuged at 4°C 1,000 × *g* for 10 min. The upper phase was removed, and the lower phase was dried with vacuum centrifuge. In this assay, the samples (100 µl), standards (100 µl), and stabilizer (100 and 150 µl) were added to 96 wells coated with IgG antibody specific to free melatonin. A solution of a biotin-labeled melatonin tracer (50 µl) was also added to the plate, and it was sealed. The plate was incubated for 1 h at 500 rpm and room temperature. Under these conditions, the specific antibody binds to melatonin in the sample or to the tracer. The plate was washed three times with 400 µl of wash solution after the incubation. The wells were empty after the wash procedure. Furthermore, 200 µl of melatonin conjugate was added into all wells, except the blank wells, which binds to the biotinylated tracer. The plate was sealed and incubated for 30 min at 500 rpm and room temperature. The plate was washed as detailed previously. Then, 200 µl of TMB substrate was added into each well. The plate was sealed and incubated for 30 min at 500 rpm and room temperature. Under these conditions, blue color is generated. Moreover, 50 µl of stop solution was added to stop the substrate reaction. The color was changed to yellow, and it was measured at 450 nm. The standard curve was plotted, and the concentrations were calculated with MARS software as mentioned in the protocol book.

##### 2.3.2.11 Determination of GSS

The concentration of GSS was measured by a commercially available ELISA kit (kit number: MBS9373764; MyBioSource, Inc. San Diego, CA, USA). The reagents were prepared as mentioned in the protocol book. Then, 50 µl of standards, samples, and blank, respectively, was added into the wells in duplicate. Horseradish peroxidase–conjugate reagent (50 µl) was added to each well. The plate was covered with a closure plate membrane and was incubated for 60 min at 37°C. The incubation mixtures of the wells were dumped into the proper waste container. Then, 350 µl of wash solution was added into each well, and after 1 min, the solution was aspirated. This step was repeated three more times for a total of four washes. Next, 50 µl of chromogen A and chromogen B, respectively, was added to each well. The plate was sealed and incubated for 15 min at 37°C. Then, 50 µl of stop solution was added to each well, and the optical density (OD) was measured at 450 nm. The standard curve was plotted, and the concentrations were calculated with MARS software as mentioned in the protocol book.

##### 2.3.2.12 Determination of IAA

The concentration of IAA was measured with a commercially available ELISA kit ((kit number: CEA737Ge; Cloud-Clone Corp., Houston, TX, USA). All reagents and solutions were prepared as described in the protocol book. Then, 50 µl of standards, samples, and blank, respectively, was added into the determined wells, and then 50 µl of detection reagent A was added to each well immediately. The plate was sealed, shaken gently, and incubated for 1 h at 37°C. The solution in the wells was aspirated. The wells were washed with 350 µl of wash solution three times. Then, 100 µl of detection reagent B working solution was added to each well. The plate was sealed and incubated for 30 min at 37°C. The wash procedure was repeated for a total of five times as described previously. Moreover, 90 µl of substrate solution was added to each well. The plate was sealed and incubated for 10 min at 37°C. Then, 50 µl of stop solution was added to each well, and the OD was measured at 450 nm. The standard curve was plotted, and the concentrations were calculated with MARS software as mentioned in the protocol book.

### 2.4 Statistical analyses

The morphological parameters and chlorophyll content data of plantlets were analyzed by one-way ANOVA followed by Tukey’s test at *p*< 0.05 using SPSS for Windows software (SPSS^®^, version 21.0). The antioxidant parameters were analyzed by one-way ANOVA followed by Tukey’s test (at *p*< 0.05) for multiple comparisons using GraphPad Prism (version 9), and the results are presented as mean ± SEM. The correlation between biochemical parameters was analyzed by Pearson correlation (*p*< 0.05).

## 3 Results

### 3.1 After-effects of ultrasonication on the growth parameters and chlorophyll content of *in vitro* potato plantlets

The morphological parameters and chlorophyll content of *in vitro* shoots were measured 4 weeks after ultrasonication, at the end of the subculture. Except for the number of nodes per plantlet (NN), which was not affected by the ultrasound (US) treatments, all other measured morphological parameters and the chlorophyll content of the 4-week-old plantlets were modified in response to ultrasonication by the end of the subculture (**Table 1**). The length of shoots (SL) decreased after ultrasonication; however, with increase in the exposure time to 30 min, the decrease was significantly lower than after the shorter exposure time (20 min). The length of the roots and the fresh weight of shoots were increased by ultrasonication but independently of the exposure time. However, the fresh weight of roots (RFW) was increased only after a shorter exposure time (20 min), while extending the exposure time of ultrasound treatment did not cause a significant change in the RFW compared with the control.

**Table 1:** Morphological parameters and chlorophyll (*chl*) content of *in vitro* plantlets 4 weeks after exposure to different ultrasound (US) treatments.

	Control^a^ (non-ultrasonicated)	20-min US^a^	30-min US
Shoot length (mm)	74.11 ± 1.53 a	59.15 ± 1.77 c	67.44 ± 1.69 b
Number of nodes/plantlets	6.91 ± 0.09 a	6.84 ± 0.14 a	7.09 ± 0.35 a
Shoot weight (g/vessel)	3.96 ± 0.27 b	5.09 ± 0.16 a	5.38 ± 0.18 a
Root length (mm)	48.97 ± 1.29 b	58.38 ± 2.12 a	58.83 ± 1.99 a
Root weight (g/vessel)	2.84 ± 0.28 b	4.79 ± 0.23 a	3.27 ± 0.25 b
*chl a* content (µg/g FW)	840.1 ± 68.8 a	677.7 ± 31.7 b	439.7 ± 38.6 c
*chl b* content (µg/g FW)	232.9 ± 18.3 a	155.7 ± 7.9 b	97.5 ± 8.5 c
*chl a* + *chl b* content (µg/g FW)	1,074.9 ± 87.1 a	834.9 ± 39.6 b	538.0 ± 42.3 c
*chl a*/*chl b*	3.60 ± 0.05 c	4.36 ± 0.04 b	4.52 ± 0.11 a

a Data from control and 20-min US were presented in a recent publication (Teixeira da Silva et al., 2020) in another context. Mean values (± standard error) followed by different letters in each row indicate significantly (P< 0.05) different values between treatments according to ANOVA followed by the Tukey test.

FW, fresh weight.

Both the *chl a* and the *chl b* contents decreased after ultrasonication. The extension of the exposure time caused a significant further decrease in both parameters. The extent of the decrease was more pronounced in *chl b* than in *chl a*. The *chl a* content was reduced by 20 and 33% after exposure to ultrasound for 20 and 30 min, respectively, compared with the control, while the decrease of *chl b* was 48 and 58% after each respective US treatment (**Table 1**).

### 3.2 Changes in the concentration of small molecule antioxidants and the activity of antioxidant pathway enzymes, melatonin, and IAA levels

**Figure 1:**
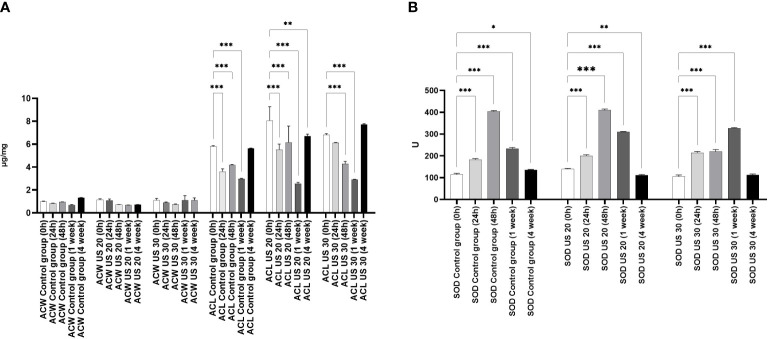
Effect of the sampling time and ultrasound treatments on the water soluble antioxidant capacity (ACW) and the lipid-soluble antioxidant capacity (ACL) **(A)** and the SOD activity **(B)**. *, **, *** mean the significant differences at P < 0.05, P < 0.01, P < 0.001, respectively.

Comparing the water-soluble antioxidant capacity (ACW) and the lipid-soluble antioxidant capacity (ACL) of control plants (**Figure 1**), we found that ACL was a multiple of ACW. Significant differences were noted only in ACL values which were similar to those of a former study that included another ultrasound treatment (Dobránszki et al., 2017). In the intensive growth phases, in 24-h- and 1-week-old plantlets, the metabolic stress triggered by growth and development was so significant that the ACL values largely decreased. The US treatments for 20 and 30 min did not influence this tendency, and a large amount of prooxidant was produced because of the intensive anabolic processes, which largely decreased the antioxidant capacity; however, the applied stress factor did not decrease it. It cannot be seen that the degradation of molecules, which eliminated prooxidants caused by metabolic and abiotic stress, was composed additively (**Figure 1**).

By studying the SOD activity (**Figure 1**), we found that it increased significantly at 24 and 48 h compared with 0 h in the control group. In 1-week-old plantlets, the SOD activity decreased, and then a further decrease could be seen in the 4-week-old plantlets. This tendency did not change significantly if the plant material was ultrasonicated for 20 min. However, in 48-h-old plants, the SOD activity did not increase in response to the 30-min-long US treatment. A significant increase of SOD activity could be detected in 1-week-old plantlets, but it did not change significantly in 4-week-old samples.

**Figure 2:**
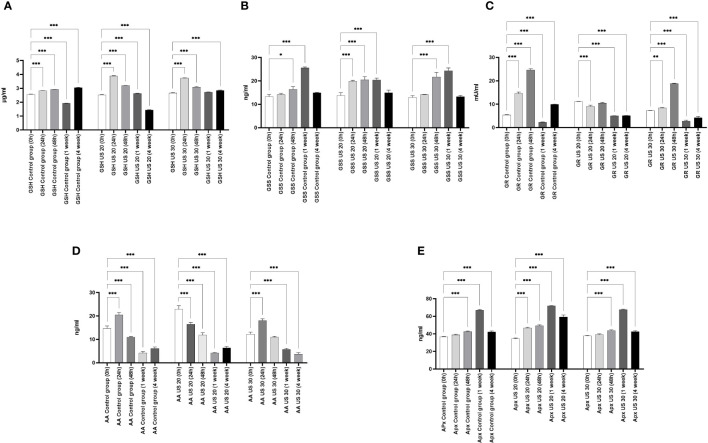
Effect of the sampling time and ultrasound treatments on reduced glutathione (GSH) concentrations **(A)**, glutathione synthetase (GSS) concentration **(B)**, glutathione reductase (GR) activity **(C)**, ascorbic acid (AA) concentration **(D)**, and ascorbic acid peroxidase (APX) concentration **(E)**. *, **, *** mean the significant differences at P < 0.05, P < 0.01, P < 0.001, respectively.

The GSH concentration of the control plant gradually increased in certain growth phases, and then in the 1-week-old plant, it significantly decreased. In this phase, the radicle transforms into root and the axillary buds transform into leaves. Similar to our former experimental results (Dobránszki et al., 2017), GSH was needed to eliminate the intensive radicle production in this phase, which can explain the decrease of its concentration (**Figure 2**). As a result of the US treatments, its concentration increased in the different sampling times; the US treatment for 30 min also resulted in its very high concentration in the 4-week-old plant. At the background of these changes, there can be a change of activity of GSS because its activity and the amount of GSH are inverses of each other. GR is responsible for maintaining the glutathione pool by splitting the conjugated glutathione. Our results are not surprising. In the control group, it follows the change of the GSH concentration. The US treatment for 20 min did not influence the direction of the quantitative changes; however, the GR activity decreased significantly. The US treatment for 30 min, which was a strong abiotic stress, increased the GR activity 48 h after ultrasonication, but in the following growth phases, it could not increase the enzyme activity compared with the control (**Figure 2**).

Besides the glutathione pool, ascorbic acid (AA) plays an important role in maintaining the redox homeostasis both in the detoxification phase and in the regeneration phase. At 24 h, its concentration in the control group increased; after that, a gradual decrease can be seen in the following growth phases (**Figure 2**). It is very considerable that, besides the metabolic stress caused by growth and development, the applied abiotic stress, *i*.*e*., ultrasonication, did not change its concentration significantly in plants.

**Figure 3:**
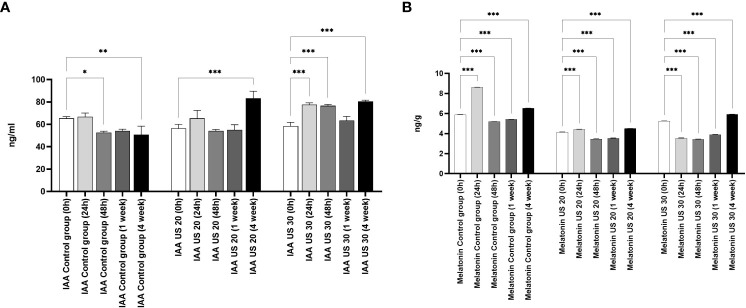
Effect of the sampling time and ultrasound treatments on the concentration of indole-3-acetic acid (IAA) **(A)**, and melatonin **(B)**. *, **, *** mean the significant differences at P < 0.05, P < 0.01, P < 0.001, respectively.

The IAA concentration did not change in the first 24 h, but in the other growth phases, it decreased in non-ultrasonicated plantlets (**Figure 3**). It did not decrease significantly in the 1-week-old plantlets compared with the initial stage. However, the US treatment produced its modulatory effect. The ultrasound treatment for 20 min significantly increased its concentration in the 4-week-old plantlets. Ultrasonication for 30 min did not cause an increase in the 1-week-old plantlets, but it caused a significant increase in all the other phases.

The MT concentration of the control group is significantly different in every growth phase (**Figure 3**). At 24 h, the MT concentration increased, then it decreased in 48-h-old and 1-week-old samples compared with the 0-h samples; in 4-week-old plantlets, however, its concentration increased significantly. Both the 20- and 30-min-long US treatments decreased the MT concentration in the plant samples compared with the control in every sampling time. After a 20-min-long ultrasonication, the MT level increased in the 24-h-old samples compared with the 0-h ones, while it decreased as a response to the 30-min-long US treatment. Both 20- and 30-min-long US treatments decreased the MT concentration in the later growth phases (48 h and 1 week), but then they increased it in 4-week-old plantlets.

## 4 Discussion

The *in vitro* plant tissue culture technique has a unique role in sustainable and competitive agriculture, horticulture, and forestry and has been successfully applied in plant breeding. Plant tissue culture has become an integral part of plant production and plant breeding. Plant tissue culture and micropropagation are playing an increasingly important role in green biotechnology. It can be an opportunity for producing excellent-quality, disease-free, and uniform plants. Various physical and chemical alternatives are searched to encourage the intensive growth of plants. Although the number of examinations is still restricted, there are evidence that ultrasonic treatment can increase the growth and development, including *in vitro* organogenesis as well (Teixeira da Silva and Dobránszki, 2014). However, there is no data on its exact mechanisms. Ultrasonication can result in cavitation and acoustic microflow, which can modify the ultrastructure of cells, enzyme activity, and cell growth. It can cause fractures in extracellular polymers, release DNA from the nucleus, decrease the stability of cells, change the permeability of the cell membrane, and modify the charges on the cell surface (Rokhina et al., 2009). It is necessary that, as a stress factor, it activates the three-level antioxidant protection system (Dobránszki et al., 2017). The ultrasonic treatment of plants also opens other routes and can be suitable for studying the role of melatonin in stress processes.

The increasing number of investigations has recently found that MT is essential in the regulation of both developmental processes and stress responses in plants (Van Tassel et al., 2001; Murch and Saxena, 2002; Arnao and Hernández-Ruiz, 2006; Wang et al., 2012; Arnao and Hernández-Ruiz, 2013; Arnao and Hernández-Ruiz, 2014; Arnao and Hernández-Ruiz, 2015; Reiter et al., 2016; Fan et al., 2018; Wang et al., 2018; Arnao and Hernández-Ruiz, 2019; Battacharya and Jha, 2020; Khan et al., 2020). The photoperiod and circadian rhythm controlling the role of MT is less relevant in plant tissue cultures than in *ex vitro* plants considering that the cultures *in vitro* are not autotroph but mixotroph (George and Davies, 2008). Our previous transcriptomic examinations (Teixeira da Silva et al., 2020) supported our present results and can give a possible explanation as to why the chlorophyll concentration decreased in response to the US treatments. Upregulation of the gene responsible for chlorophyllase was recently detected 24 h after the 20-min-long US treatment of single-node *in vitro* potato explants, which suggested the potential degradation of *chl a* and *chl b* (Teixeira da Silva et al., 2020). Our current measurements of chlorophyll content (**Table 1**) confirmed our earlier hypothesis based on a transcriptomic study (Teixeira da Silva et al., 2020) and proved that ultrasonication (35 kHz and 70 W for 20 min) by a liquid-based ultrasonicator caused the degradation of both *chl a* and *chl b*. In addition, the extension of the exposure period from 20 to 30 min led to a further decrease in the chlorophyll content of the 4-week-old plantlets in our present study. Exploring the real cause of the further decreases in the chlorophyll content by extending the exposure time of ultrasonication requires further investigation, but this may be due to either a further increase in the expression level of the gene responsible for chlorophyllase or alterations in the expression level of other genes responsible for the synthesis or degradation of chlorophyllase.

### 4.1 Endogenous melatonin as an antioxidant

**Figure 4:**
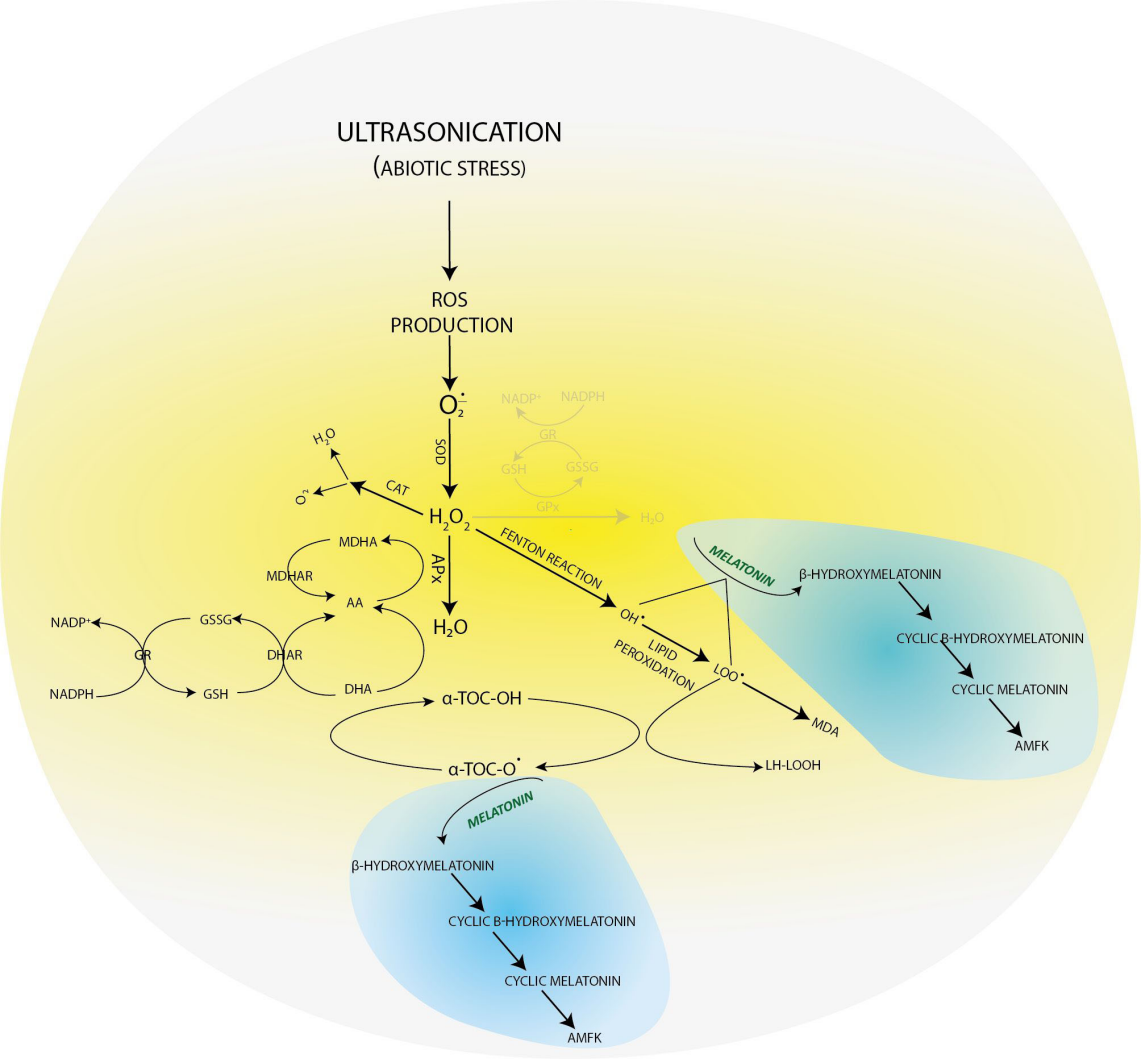
Molecular transformations of melatonin and its role in reactive oxygen species elimination. All products of its molecular transformation result in strong antioxidants (in blue bubbles), and they, as radical catchers, eliminate free electrons after exposure of the plant to different stresses.

In our previous experimental work (Dobránszki et al., 2017), when we applied a gentler US treatment, the results showed that the three-level antioxidant system was activated for the elimination of prooxidants. GSH was available for the glutathione-dependent enzymes of the enzymatic route. The GSH concentration increased for the effect of stress (for example, in the 4-week-old plants, it was 25 uM/ml) compared with the control group (**Figure 2**). AA, essential for GSH regeneration, was 40 ng/ml in the 4-week-old plantlets. If a much stronger ultrasound stress was applied (Dobránszki et al., 2020), as presented in this work, it could badly disturb the membrane integrity according to the literature (Rokhina et al., 2009), but we have got unexpected plant responses. The concentration of small molecule antioxidants was not increased compared with the control plants. These results suggest that, although the antioxidant protection system was activated, the small molecule antioxidants could not adjust the prooxidant–antioxidant balance. Therefore, another protection system was needed, which helps in eliminating the lipid peroxidation processes and supports the plant for it not to die from stress (**Figure 4**).

As was recently investigated, the gene expression of some enzymes related to the biosynthesis of MT and IAA changed when single-node *in vitro* explants of potato were exposed to ultrasonication for 20 min (Teixeira da Silva et al., 2020). Immediately after the 20-min-long US treatment, the genes for tryptophan (Trp) synthase (EC 4.2.1.20) were upregulated, causing an increased level of Trp, as a direct consequence of the US treatment. Trp is the precursor of both IAA and MT biosynthesis (Posmyk and Janas, 2009; Fan et al., 2018). Its downregulation was detected only 48 h after a 20-min-long ultrasonication. The measured MT level of the ultrasonicated samples, independently of the exposure time, was lower at 24 h, 48 h, and 1 week after ultrasonication compared with the control, presumably due to its use in stress mitigation caused by ultrasonication. However, by the end of the subculture, the MT level was significantly the highest after a 20-min-long ultrasonication, while its level remained significantly lower than in the control when ultrasonication was carried out for 30 min. However, the experimental results show no increase in MT levels after US treatment except for the 4-week-old plant but rather an increase in IAA concentration. This suggests that an increase in the expression of the genes for Trp synthase confirms the pathway for IAA synthesis, and the endogenous MT levels are generally constant and low in potato plants grown *in vitro*.

In plant tissue cultures, the low yield of the secondary metabolites can be explained with the lack of cell differentiation (Verpoorte et al., 2002). As a consequence, the concentrations of both the lipid-soluble (ACL) and especially the water-soluble antioxidants (ACW) are very low. However, the MT concentration was constant, which referred to its important role in maintaining the redox homeostasis during plant growth. The applied US treatment decreased its concentration, which can be explained by the fact that MT is an amphipathic molecule and can directly eliminate free radicals. Unlike the other radical catchers, it is a multifunctional antioxidant; it can easily get through the cell membranes because of its high lipophilicity and hydrophilicity (Hardeland, 2016). Unlike the other small-molecule biological antioxidants such as ascorbic acid, α-tocopherol, lipoic acid, *etc*., MT does not go through a redox cycle; it can be considered a terminal antioxidant. It suffers a molecular transformation while efficiently eliminating free electrons from the system. All the products of this transformation are also strong antioxidants themselves (for example, cyclic 3-hydroxi-melatonin, 2-formil-5-methoxyquinuramine, and *N*-acetil-5-methoxyquinuramine) (Davanipour et al., 2009). These metabolites (**Figure 4**) work as radical catchers sometimes more aggressively than the MT itself regarding ROS neutralization. Besides this, most of these processes have one more ROS in every step, so one MT molecule can bond 10 ROS, in contrast with the classical antioxidants. It was found that MT promotes to repair the oxidized DNA. This can be due to the fact that MT can transform the guanosine radical into guanosine with electron transfer. It was shown that MT decreased the production of 8-hydroxi-2′-dezoxiguanosine, a damaged DNA product, which is 60–70 times efficient than certain classical antioxidants (ascorbic acid and α-tocopherol) (Qi et al., 2000; Reiter et al., 2016). The synthesis of superoxide anions mainly takes places in mitochondria during the oxidative phosphorylation. The superoxide anions quickly enzymatically transform into H_2_O_2_ for the effect of SOD. Then, H_2_O_2_ transforms into water or a very poisonous hydroxyl radical. Although hydroxyl radical formation can take place in many ways; the *in vivo* degradation of superoxide anion and hydrogen peroxide catalyzed by transition metal is the most important mechanism. Hydroxyl radicals form from hydrogen peroxide during the oxygen metabolism of the cell and the Fenton and the Haber–Weiss reactions in the presence of free iron and copper ions (Manchester et al., 2015). OH• forms in the Fenton reaction, when hydrogen peroxide interacts with the transition metals (Fe^2+^, Cu^1+^, *etc*.).

**Figure 5:**
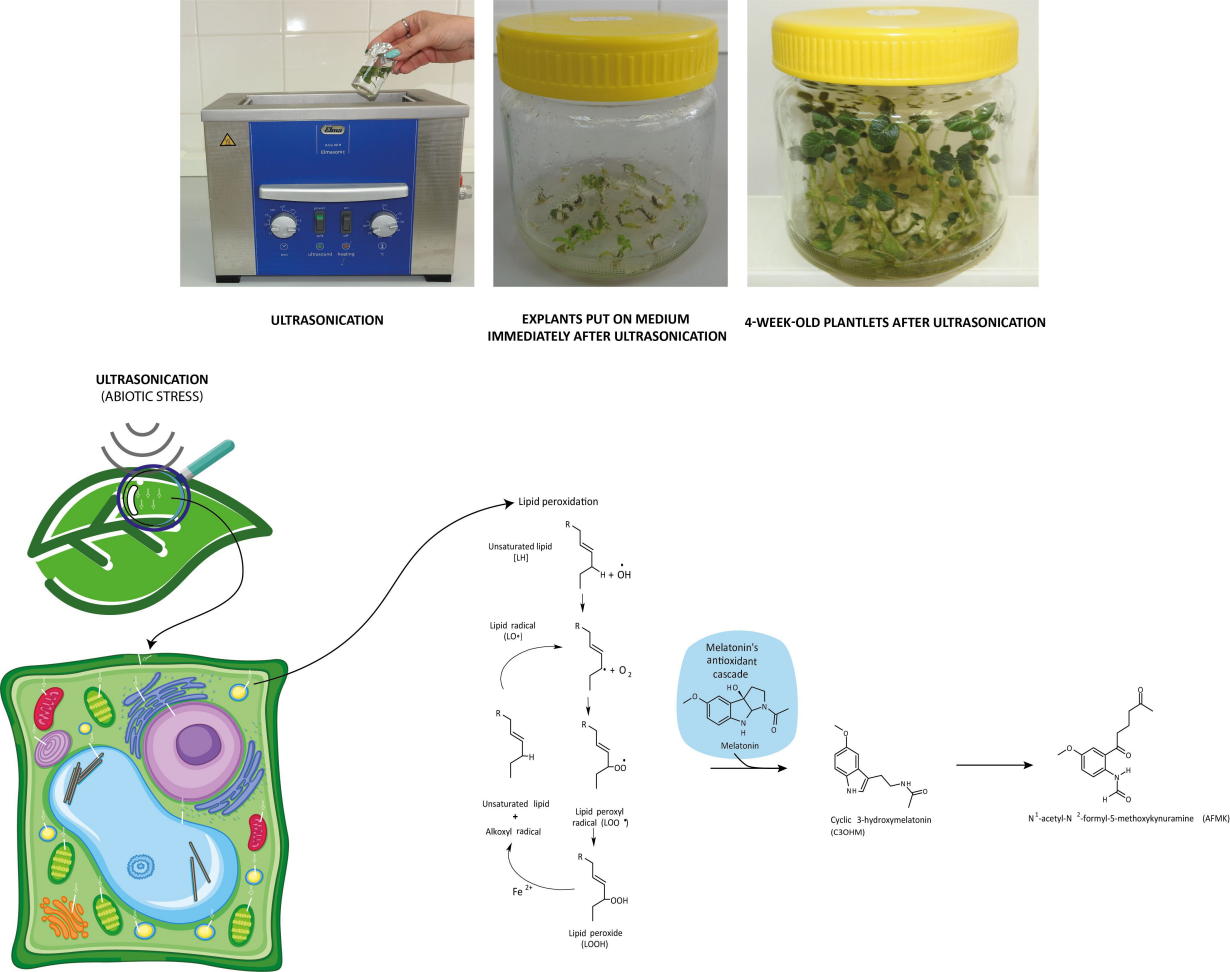
Ultrasonication of *in vitro* single-node potato explants and role of melatonin in the recovery of redox homeostasis after lipid peroxidation triggered by ultrasonication.

If in certain growth cycles only metabolic stress affected the plant, the MT level correlated to the IAA level. The prooxidants, which form on the biosynthetic routes, were eliminated by the redox system from plant cells. If abiotic stress, the ultrasonication, which changes the integrity of the membrane system, was applied, the level of the measurable MT isomer decreased because hydroxy-melatonin derivatives possibly form. These compounds are used for eliminating the hydrogen peroxides which form from the superoxide anion in enzymatic processes during the relief of the redox system. It can be seen that the MT level negatively correlated with the redox enzymes and glutathione. The severe US treatment (30-min-long ultrasonication) did not cause very drastic changes in the redox homeostasis of the plants, which could not have been compensated (**Figure 5**).

### 4.2 Endogenous melatonin as a growth regulator

The gene expression of indole pyruvate decarboxylase (EC 4.1.1.74), the key enzyme of the IAA biosynthesis (Koga, 1995), was upregulated 24 h after exposure to US for 20 min. At the same time (24 h), the expression intensity of tryptophan-tRNA ligase (EC 6.1.1.2) producing L-tryptophanyl-tRNA from Trp significantly decreased. Their downregulation was detected only 1 and 4 weeks after ultrasonication, respectively (Teixeira da Silva et al., 2020). This suggested that the 20-min-long ultrasonication supports the biosynthesis of IAA 24 h after the US treatment. Although data on the morphological parameters, like increasing fresh weight of shoots and roots and root length, may refer to auxin predominance in plantlets, the exact measurements of IAA content did not show a significantly increased level of IAA compared with the control after a 20-min-long exposure time of US. However, when the explants were treated with ultrasound for 30 min, the IAA level significantly increased 48 h and 1 week after ultrasonication.

The decrease in the shoot length of 4-week-old plantlets after ultrasonication of explants for 20 min may be connected to the earlier results that came from a transcriptomic study (Teixeira da Silva et al., 2020). In this study, the upregulation of the gene coding for gibberellin 2β-dioxygenase (EC 1.14.11.13) was detected 24 h after ultrasonication, which could result in catabolites of gibberellic acid. The SL value of the plantlets significantly increased after a 30-min-long ultrasonication compared with the shorter US treatments but remained significantly lower than that of the control. The background processes of these results require further investigations.

It is long known that the different phytohormones have a significant role in every aspect of plant physiology during growth and development. The effects of the application of cytokinin, GA_3_, ABA, auxin, brassinosteroides, and jasmonic acid on plants have been revealed. Tryptophan is the frequent precursor of the biosynthesis of MT and IAA (Posmyk and Janas, 2009; Erland et al., 2015; Erland and Saxena, 2018; Khan et al., 2020), which may result in these compounds having similar regulatory functions in plant growth and development (Shi et al., 2015). The connections between MT and a lot of other hormones have directly and indirectly been studied, but few signal transmission models were suggested in the adaptation to US stress as to how it protects photosynthesis with chlorophyll synthesis. In contrast with other long-studied abiotic stresses, our present investigation confirmed that US stress does not decrease the IAA concentration in plants; rather, it increases it (Shakeel et al., 2021).

Another indirect effect of MT is connected to the regulation of the activity of antioxidant enzymes, like GPX, GR, SOD, and CAT, and it is able to stimulate the synthesis of GSH (Rodriguez et al., 2004; Erland and Saxena, 2018). Taking into account its multifunctionality in plants, melatonin has recently been considered to be not only a plant hormone but also a master regulator in plants (Arnao and Hernández-Ruiz, 2019).

**Figure 6:**
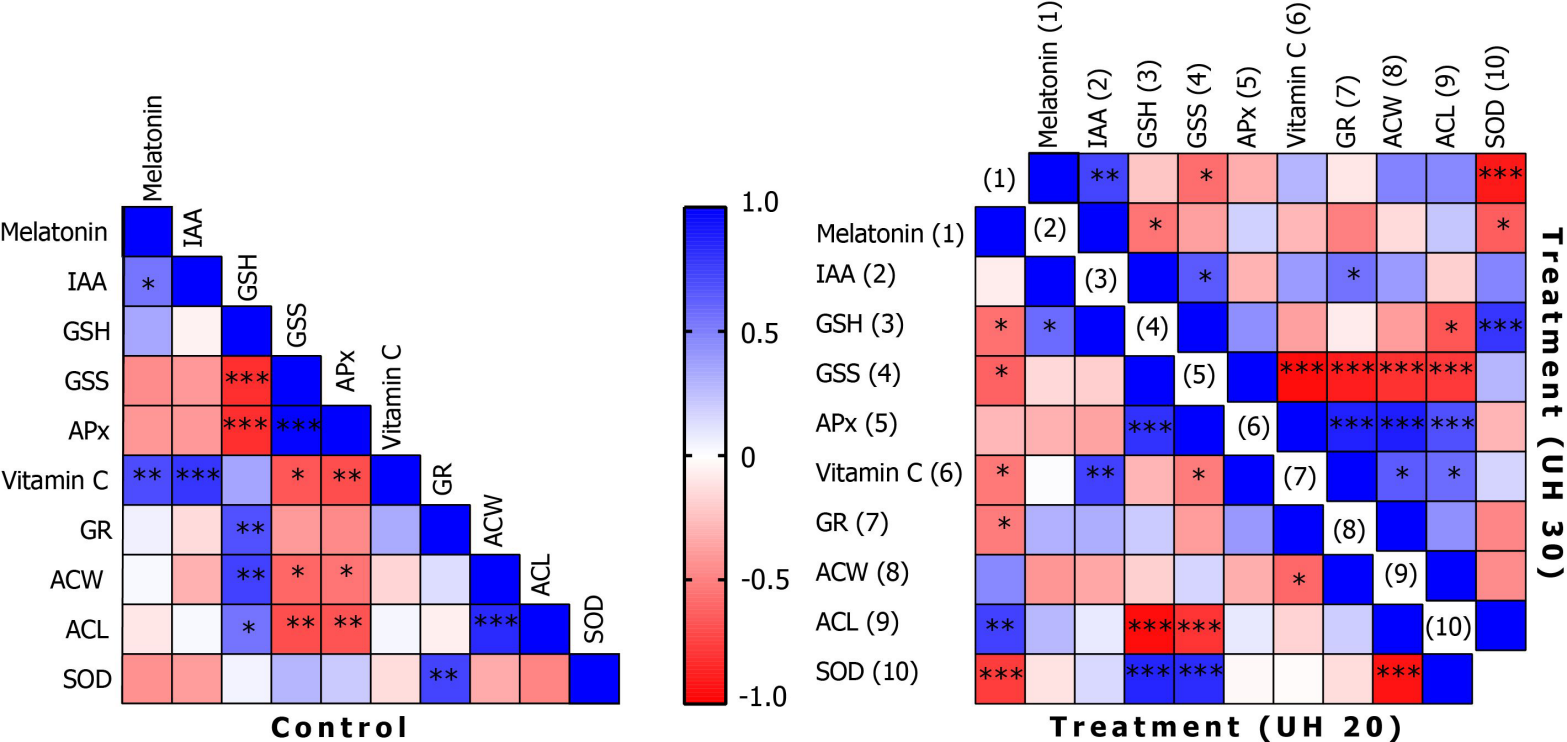
Correlations of biochemical parameters in the control group and after ultrasonication for 20 min (treatment, UH 20) and 30 min (treatment, UH 30), respectively. *, **, *** mean the significant differences at P < 0.05, P < 0.01, P < 0.001, respectively.

Concentrations of endogenous MT correlate with IAA levels (**Figure 6**). In addition, it shows a positive correlation with AA levels. The IAA levels also correlate positively with AA levels. Although there are common biosynthetic pathways for MT and IAAs in plants, the amount and the extent of their synthesis may be also determined by the concentration of AA. The question arises as to whether AA may have a regulatory role in the synthesis of tryptophan-dependent IAA formed by the other tryptophan-independent pathway. After 20 min of US treatment, MT shows a negative correlation at several points in the antioxidant system, including the concentration of GSH or SOD enzyme activity among the small-molecule antioxidants in addition to AA. A strong positive relationship was found with the amount of ACL. This suggests that MT and the MT derivatives formed during its catabolism strengthen the group of compounds with lipid-soluble antioxidant capacity. This does not change even if the stronger US treatment was applied. It is possible that MT directs the elimination of excess H_2_O_2_ by increasing the activity of SOD and by the synthesis of AA. Similar results were obtained by Kostopoulou et al. (2015) when exogenous MT and AA treatment was applied in citrus fruits.

## 5 Conclusion

MT appears to play a clear role in plant abiotic or metabolic stress adaptation in collaboration with other plant hormones. Its association with ROS provides evidence that MT is a key component at the center of the redox network from which various biochemical, cellular, and physiological responses are regulated. MT-ROS are self-regulating through the regulation of directly interacting systems and the biosynthesis of their own biosynthesis and catabolic genes. There is a close relationship between the plant hormone IAA and MT and AA. MT has a dual role in plants, acting both as an antioxidant and a growth regulator. It would also be important to examine how the activity of the redox system changes in different photoperiods and whether MT has a regulatory role in activating the system itself.

## Data availability statement

The original contributions presented in the study are included in the article/Supplementary Material. Further inquiries can be directed to the corresponding author.

## Author contributions

JD and JR devised the experimental design. JD conducted the ultrasonication experiments as well as collected and evaluated the growth data. GP-A, IF, and PM-B conducted the biochemical analyses under the supervision of JD and JR. All authors analyzed the data, wrote or contributed to all drafts of the manuscript, and take public responsibility for the data. All authors contributed to the article and approved the submitted version.

## Funding

Project no. TKP2021-EGA-20 (Biotechnology) has been implemented with the support provided from the National Research, Development, and Innovation Fund of Hungary, financed under the TKP2021-EGA funding scheme.

## Conflict of interest

The authors declare that the research was conducted in the absence of any commercial or financial relationships that could be construed as a potential conflict of interest.

## Publisher’s note

All claims expressed in this article are solely those of the authors and do not necessarily represent those of their affiliated organizations, or those of the publisher, the editors and the reviewers. Any product that may be evaluated in this article, or claim that may be made by its manufacturer, is not guaranteed or endorsed by the publisher.

## References

[B1] Arnao M. B. Hernández-Ruiz J. (2006). The physiological function of melatonin in plants. Plant Signal. Behav. 1 (3), 89–95. doi: 10.4161/psb.1.3.2640 19521488 PMC2635004

[B2] Arnao M. B. Hernández-Ruiz J. (2013). Growth conditions determine different melatonin levels in *Lupinus albus* l. J. Pineal Res. 55, 149–155. doi: 10.1111/jpi.12055 23600673

[B3] Arnao M. B. Hernández-Ruiz J. (2014). Melatonin: plant growth regulator and/or biostimulator during stress? Trends Plant Sci. 19, 789–797. doi: 10.1016/j.tplants.2014.07.006 25156541

[B4] Arnao M. B. Hernández-Ruiz J. (2015). Functions of melatonin in plants: A review. J. Pineal Res. 59, 133–150. doi: 10.1111/jpi.12253 26094813

[B5] Arnao M. B. Hernández-Ruiz J. (2019). Melatonin: A new plant hormone and/or a plant master regulator? Trends Plant Sci. 24 (1), 38–48. doi: 10.1016/j.tplants.2018.10.010 30446305

[B6] Battacharya P. Jha S. (2020). “Melatonin: An alternative signal to antioxidant enzyme modulation in plants,” in Neurotransmitters in plant signaling and communication, sinaling and communication in plants. Ed. Baluška F. Mukherjee S. Ramakrishna A., (Cham: Springer). pp 241–251.

[B7] Cassone V. M. (1998). Melatonin’s role in vertebrate circadian rhythms. Chronobiol. Int. 15 (5), 457–473. doi: 10.3109/07420529808998702 9787936

[B8] Davanipour Z. Poulsen H. E. Weimann A. Sobel E. (2009). Endogenous melatonin and oxidatively damaged guanine in DNA. BMC Endocr. Disord. 9, 22. doi: 10.1186/1472-6823-9-22 19835624 PMC2771025

[B9] Dijk D. J. Czeisler C. A. (1994). Paradoxical timing of the circadian rhythm of sleep propensity serves to consolidate sleep and wakefulness in humans. Neurosci. Lett. 166 (1), 63–68. doi: 10.1016/0304-3940(94)90841-9 8190360

[B10] Dobránszki J. Asbóth G. Homoki D. Bíró-Molnár P. Teixeira da Silva J. A. Remenyik J. (2017). Ultrasonication of *in vitro* potato single node explants: Activation and recovery of antioxidant defence system and growth responses. Plant Physiol. Biochem. 121, 153–160. doi: 10.1016/j.plaphy.2017.10.022 29102903

[B11] Dobránszki J. Hidvégi N. Gulyás A. Tóth B. Teixeira da Silva J. A. Dobránszki J. (2020). Abiotic stress elements in *in vitro* potato (*Solanum tuberosum* l.) exposed to air-based and liquid-based ultrasound: A comparative transcriptomic assessment. Prog. Biophysics Mol. Biol. 1559 (158), 47–56. doi: 10.1016/j.pbiomolbio.2020.09.001 32916176

[B12] Dobránszki J. Mendler-Drienyovszki N. (2014). Cytokinin-induced changes in the chlorophyll content and fluorescence of *in vitro* apple leaves. J. Plant Physiol. 171, 1472–1478. doi: 10.1016/j.jplph.2014.06.015 25108261

[B13] Erland L. A. Murch S. J. Reiter R. J. Saxena P. K. (2015). A new balancing act: The many roles of melatonin and serotonin in plant growth and development. Plant Signal. Behav. 10, e1096469. doi: 10.1080/15592324.2015.1096469 26418957 PMC4883872

[B14] Erland L. A. E. Saxena P. (2018). Auxin driven indoleamine biosynthesis and the role of tryptophan as an inductive signal in hypericum perforatum (L.). PloS One 14 (10), e0223878. doi: 10.1371/journal.pone.0223878 PMC6797091 31622392

[B15] Fan J. Xie Y. Zhang Z. Chen L. (2018). Melatonin: A multifunctional factor in plants. Int. J. Mol. Sci. 19, 1528. doi: 10.3390/ijms19051528 PMC5983796 29883400

[B16] George E. F. Davies W. (2008). “Effects of physical environment,” in Plant propagation by tissue culture 3rd edition, vol. 1 . Eds. George E. F. Hall M. A. De Klerk G.-J. (Dordrecht, The Nederlans: Springer), 423–464.

[B17] Hardeland R. (2016). Melatonin in plants – diversity of levels and multiplicity of functions front. Plant Sci. 7. doi: 10.3389/fpls.2016.00198 PMC4759497 26925091

[B18] Khan A. Numan M. Khan A. L. Lee I.-J. Imran M. Asaf S.. (2020). Melatonin: Awakening the defense mechanisms during plant oxidative stress. Plants 9, 407. doi: 10.3390/plants9040407 PMC7238205 32218185

[B19] Koga J. (1995). Structure and function of indolepyruvate decarboxylase, a key enzyme in indole-3-acetic acid biosynthesis. Biochim. Biophys. Acta 1249, 1–13. doi: 10.1016/0167-4838(95)00011-i 7766676

[B20] Kostopoulou Z. Therios I. Roumeliotis E. Kanellis A. K. Molassiotis A. (2015). Melatonin combined with ascorbic acid provides salt adaptation in *Citrus aurantium* l. seedlings. Plant Physiol. Biochem. 86, 155–165. doi: 10.1016/j.plaphy.2014.11.021 25500452

[B21] Li H. Chang J. Chen H. Wang Z. Gu X. Wei C.. (2017). Exogenous melatonin confers salt stress tolerance to watermelon by improving photosynthesis and redox homeostasis. Front. Plant Sci. 8. doi: 10.3389/fpls.2017.00295 PMC5331065 28298921

[B22] Manchester L. C. Coto-Montes A. Boga J. A. Andersen L. P. H. Zhou Z. Galano A.. (2015). Melatonin: An ancient molecule that makes oxygen metabolically tolerable. J. Pineal Res. 59 (4), 403–419. doi: 10.1111/jpi.12267 26272235

[B23] Murashige M. Skoog F. (1962). A revised medium for rapid growth and bioassay with tobacco tissue culture. Physiol. Plant 15, 473–497. doi: 10.1111/j.1399-3054.1962.tb08052.x

[B24] Murch S. J. Saxena P. K. (2002). Melatonin: a potential regulator of plant growth and development? Vitro Cell. Dev. Biol. Plant 38, 531.

[B25] Posmyk M. M. Janas K. M. (2009). Melatonin in plants. Acta Physiol. Plant 31, 1. doi: 10.1007/s11738-008-0213-z

[B26] Qi W. Reiter R. J. Tan D. X. Manchester L. C. Siu A. W. Garcia J. J. (2000). Increased levels of oxidatively damaged DNA induced by chromium(III) and H_2_O_2_: protection by melatonin and related molecules. J. Pineal Res. 29 (1), 54–61. doi: 10.1034/j.1600-079x.2000.290108.x 10949541

[B27] Reiter R. J. Mayo J. C. Tan D.-X. Sainz R. M. Alatorre-Jimenez M. Qin L. (2016). Melatonin as an antioxidant: Under promises but over delivers. J. Pineal Res. 61, 3, 253–278. doi: 10.1111/jpi.12360 27500468

[B28] Rodriguez C. Mayo J. C. Sainz R. M. Antolin I. Herrera F. Martin V.. (2004). Regulation of antioxidant enzymes: A significant role for melatonin. J. Pineal Res. 36, 1–9. doi: 10.1046/j.1600-079x.2003.00092.x 14675124

[B29] Rokhina E. V. Lens P. Virkutyte J. (2009). Low-frequency ultrasound in biotechnology: State of the art. Trends Biotechnol. 27, 298–306. doi: 10.1016/j.tibtech.2009.02.001 19324441

[B30] Shakeel A. Kamran M. Zhou X. Irshad A. Meng X. Javed T.. (2021). Melatonin improves the seed filling rate and endogenous hormonal mechanism in grains of summer maize. Physiol. Plantarum. 172 (2), 1059–1072. doi: 10.1111/ppl.13282 33206390

[B31] Shi H. Jiang C. Ye T. Tan D.-X. Reiter R. J. Zhang H.. (2015). Comparative physiological, metabolomic, and transcriptomic analyses reveal mechanisms of improved abiotic stress resistance in bermudagrass [Cynodon dactylon (L). pers.] by exogenous melatonin. J. Exp. Bot. 66 (3), 681–694. doi: 10.1093/jxb/eru373 25225478 PMC4321537

[B32] Teixeira da Silva J. A. Dobránszki J. (2014). Sonication and ultrasound: Impact on plant growth and development. Plant Cell. Tissue Organ Cult. (PCTOC) 117 (2), 131–143. doi: 10.1007/s11240-014-0429-0

[B33] Teixeira da Silva J. A. Hidvégi N. Gulyás A. Tóth B. Dobránszki J. (2020). Transcriptomic response of *In vitro* potato (*Solanum tuberosum* l.) to piezoelectric ultrasound. Plant Mol. Biol. Rep. 38, 404–418. doi: 10.1007/s11105-020-01204-3

[B34] Van Tassel D. L. Roberts N. Lewy A. O’neill S. D. (2001). Melatonin in plant organs. J. Pineal Res. 31, 8–15. doi: 10.1034/j.1600-079x.2001.310102.x 11485009

[B35] Verpoorte R. Contin A. Memelink J. (2002). Biotechnology for the production of plant secondary metabolites. Phytochem. Rev. 1, 13–25. doi: 10.1023/A:1015871916833

[B36] Wang Y. Reiter R. J. Chan Z. (2018). Phytomelatonin: a universal abiotic stress regulator. J. Exp. Bot. 69 (5), 963–974. doi: 10.1093/jxb/erx473 29281056

[B37] Wang P. Yin L. Liang D. Li C. Ma F. Yue Z. (2012). Delayed senescence of apple leaves by exogenous melatonin treatment: Toward regulating the ascorbate-glutathione cycle. J. Pineal Res. 53, 11–20. doi: 10.1111/j.1600-079X.2011.00966.x 21988707

